# Benign Recurrent Sixth Nerve Palsy in a Child

**DOI:** 10.1155/2017/8276256

**Published:** 2017-12-28

**Authors:** Rita Gonçalves, Pedro Coelho, Carlos Menezes, Isabel Ribeiro

**Affiliations:** ^1^Department of Ophthalmology, Hospital Pedro Hispano, Matosinhos, Portugal; ^2^Department of Ophthalmology, Hospital de Santa Luzia, Viana do Castelo, Portugal

## Abstract

Benign recurrent sixth nerve palsy in children is rare. It typically occurs following viral illness or immunization, and prognosis is usually excellent. However, it is always a diagnosis of exclusion given the more serious alternative causes. Therefore, a thorough examination with brain imaging is recommended. The authors report a child with six recurrent episodes of isolated benign sixth nerve palsy.

## 1. Introduction

Similar to adults, isolated sixth (6th) nerve palsy is the most common cranial nerve palsy in the pediatric population [1]. However, it is far less frequent in children and demands an immediate and thorough investigation, due to potentially devastating common causes in these ages, such as trauma, neoplasms, central nervous system infection, or raised intracranial pressure [2, 3]. Benign causes account for just 9 to 14% of all 6th nerve palsies in children [4]. A recurrent benign form of 6th nerve palsy, a rarer still palsy, has been described in the literature, and it is of presumed inflammatory etiology, associated with live attenuated vaccines, or following viral and bacterial infections such as* Varicella zoster*, Epstein-Barr virus, Cytomegalovirus, or* Coxiella burnetii *[5, 6].

We report a case of a child with six self-limited documented episodes of isolated benign 6th nerve palsy.

## 2. Case Report

A previously healthy four-year-old girl was presented to our emergency room with complaints of binocular horizontal diplopia of sudden onset and strabismus. Ophthalmological examination revealed an esotropia of the left eye in primary position, with marked abduction deficit, no palpebral fissure changes, and a vicious position of the head (left head turn). Visual acuity was normal and was no significant refractive error on cycloplegic refraction (OD −0.50 − 0.50 × 10°, OE −0.25 − 0.50 × 170°) for her age. Fundus examination was normal with no evidence of papilledema. One week prior to the event, the child had a history of fever and productive cough, and she was under treatment with amoxicillin. There was no history of live attenuated vaccine administration in the previous days. At admission, she was apyretic and hemodynamically stable and without any signs of active infectious disease. Neurological examination was unremarkable except the cranial nerve palsy.

From the additional workout, a lumbar puncture was performed, with a normal opening pressure, and cerebrospinal fluid (CSF) analysis revealed normal features. Urgent magnetic resonance imaging (MRI) and angiography of brain were performed and demonstrated the absence of intracranial and orbital lesions.

A diagnosis of isolated left 6th nerve palsy was made and, in order to obtain symptomatic relief, 24-hour alternate eye patching was started.

During the entire hospitalization, she remained apyretic and without new neurological signs. No pathogens were isolated from blood and CSF cultures.

The child was discharged with indication to keep alternate eye patching to prevent amblyopia and muscular fibrosis. Two months after the initial presentation, complete and spontaneous resolution was observed, with normal oculomotor balance.

**Figure 1:**
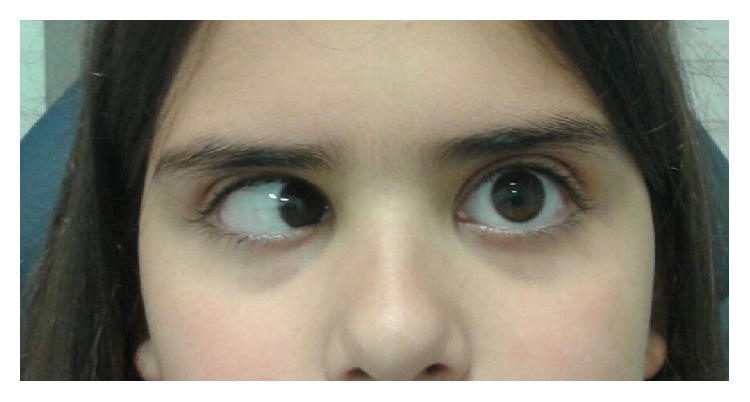
Left gaze position, showing complete left sixth nerve palsy. The child was 9 years old at the time of this photograph. Informed consent has been obtained from a parent of the child to publish this photograph.

Since the inaugural event, she has suffered five more episodes of complete left 6th nerve palsy (see Figure 1), at intervals between one and two years, the last episode being at 11 years of age. All episodes occurred following nonspecific febrile illness, except for the last two where no trigger was identified. Systemic complete workout, with neuroimaging (MRI with gadolinium) and CSF analysis, was repeated after the second episode and was revealed to be normal. Isolated 6th nerve palsy was the only neurological sign during these events. Complete resolution of the palsy with full recovery of oculomotor balance was achieved in the following weeks or first two months after the initial presentation, in all episodes.

The child is currently 11 years old and was followed up closely by the pediatric neurology and ophthalmology team. A new brain MRI with gadolinium was performed last year and revealed to be normal.

## 3. Discussion

Benign isolated 6th nerve palsy of childhood is rare, and recurrences are rarer [7]. By definition, it is not due to a threatening cause, such as an underlying intracranial lesion, and recovery is expected. It has a female predominance and the left side is most commonly affected [8], like in the case we presented.

This condition typically occurs following viral illnesses, infections, and immunization involving attenuated live vaccinations. In general, prognosis for benign recurrent 6th nerve palsy is excellent, and majority of patients recover full muscle function [6]. Failure to improve suggests more serious intracranial pathology [9].

Up to third of cases of isolated 6th nerve palsy have a neoplastic origin [10]. Therefore, a thorough history and physical examination to evaluate for any other neurological symptoms or signs, followed by a brain MR imaging, are recommended. Lumbar puncture and other investigations should be made on a case-by-case basis.

Despite a seemingly temporal association between benign 6th nerve palsy and infections in children, the exact pathophysiological mechanism remains unclear. It has been postulated to be caused by damage arising from autoimmune mediation or direct viral infection leading to demyelination or by a localised arteritis [11].

In our case, two of the six episodes of recurrence had no recognizable febrile illness, suggesting that not all benign 6th nerve palsies are due to postinfectious illness. Knapp and Gottlob [12] also reported two cases of benign recurrent 6th nerve palsy in children with no obvious etiology or any underlying precipitating factors. Alternative underlying causes may include neurovascular compression by aberrant artery and migraine [13].

A thorough investigation was carried out before starting treatment. Alternate eye patching was the chosen treatment to prevent amblyopia and allow for binocular visual function in the long term.

Long-term observation is crucial in children with recurrent 6th nerve palsy. Benign 6th nerve palsy may be after all a good surprise in the clinical spectrum of cranial nerve palsy, and it is always a diagnosis of exclusion given the more serious and life-threatening alternative causes.
